# The NTDs and Vaccine Diplomacy in Latin America: Opportunities for United States Foreign Policy

**DOI:** 10.1371/journal.pntd.0002922

**Published:** 2014-09-25

**Authors:** Peter J. Hotez

**Affiliations:** 1 Sabin Vaccine Institute and Texas Children's Hospital Center for Vaccine Development, Departments of Pediatrics and Molecular Virology and Microbiology, National School of Tropical Medicine, Baylor College of Medicine, Houston, Texas, United States of America; 2 James A. Baker III Institute for Public Policy, Rice University, Houston, Texas, United States of America; 3 Department of Biology, Baylor University, Waco, Texas, United States of America

*Recently published prevalence estimates of neglected tropical diseases (NTDs) in five Latin American countries—Bolivia, Cuba, Ecuador, Nicaragua, and Venezuela—could suggest a new direction for United States foreign policy in the region.*

In their 2008 report entitled “US–Latin America Relations: A New Direction for A New Reality,” the Council on Foreign Relations highlighted dramatic and recent shifts in US foreign policy towards Latin America [1]. Briefly stated, following a period of 150 years of US hegemony in the Latin American and Caribbean (LAC) region under the auspices of the Monroe Doctrine, increasingly, LAC nations are looking to other partners such as China and India, as well as the Middle East [1]. In parallel, favorable attitudes towards the US have dropped to unprecedented lows in many LAC countries (Figure 1) [1].

**Figure 1:**
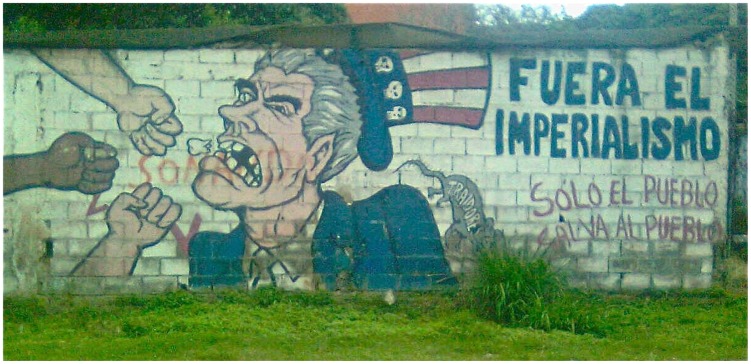
Street art in Caracas, Venezuela. Photo credit: Erik Cleves Kristensen.

Perhaps the most dramatic examples of falling US hemispheric leadership are the nation of Venezuela and its fellow leftist-aligned countries of Bolivia, Ecuador, and Nicaragua, as well as Cuba:

- **Bolivia.** According to the US Department of State, US relations deteriorated beginning in 2006 with the election of Evo Morales [2]. Bolivia expelled both the US Ambassador and US Drug Enforcement Administration (DEA) in 2008, and later the US Agency for International Development (USAID) in 2013 [2]. Most recently, the Bolivian president, along with the presidents of Nicaragua and Venezuela, granted political asylum to former CIA employee Edward Snowden [3].
- **Ecuador.** The US State Department also reports that in 2011, the Ecuadorian Government declared the US Ambassador persona non grata over public WikiLeaks disclosures, and the Ecuadorian Ambassador to the US was expelled [4]. Although high level diplomatic relations were subsequently restored, in 2013 the Foreign Minister of Ecuador provocatively declared that Latin America is not the “US backyard,” nor should the US dictate on rights and freedoms especially in the context of newly revealed US surveillance activities [3]. According to the Council on Foreign Relations both President Rafael Correa of Ecuador and Bolivian President Morales openly criticize the US Government as a means to fortify popular support at home [1].
- **Nicaragua.** During the 1980s the Reagan Administration (according to some experts) led an undeclared war against the Sandinista regime in Nicaragua [5]. Since then the US has maintained diplomatic relations through an, at times, rocky relationship, of late marred by alleged irregularities in the 2011 presidential election, which according to the US State Department represent a “setback to democracy in Nicaragua” [6].
- **Venezuela.** Under President Hugo Chavez and his successor Nicolas Maduro, Venezuela and the US have not had representation at the level of ambassador since 2010 [7]. The US State Department reports that late in 2013 the Venezuelan Government ordered the expulsion of the US charge d'affaires after declaring her persona non grata [7]. Allegedly, Mr. Maduro has also accused the US DEA of deliberately planting 1.3 tons of cocaine that was seized in 2013 from an Air France plane leaving Caracas [8].
- **Cuba.** The US broke its diplomatic relations with communist Cuba in 1961 [9].

Aside from their strong leftist governments and varying degrees of strained or severed diplomatic relationships with the US, four of these five countries—Bolivia, Ecuador, Nicaragua, and Venezuela—also stand out for their unusually high levels of poverty and disease.

The LAC region has a population of almost 600 million people [10], of whom 12 percent live in Bolivia, Cuba, Ecuador, Nicaragua, and Venezuela combined (Table 1) [11]. Of these five countries, Venezuela has by far the largest population of almost 30 million people [11]. Using an earlier estimate of 99 million people in the LAC region living on less than $2 per day [12], roughly 10 percent of the region's extremely poor people live in Bolivia, Ecuador, Nicaragua, and Venezuela [13]. Venezuela has almost 4 million people living on less than $2 per day, while 1.6–2.6 million impoverished individuals live in Bolivia, Ecuador, and Nicaragua [13].

**Table 1:** Poverty in five Latin American countries.

Country	Total Population (Year) [10], [11]	% Living on less than $2 per day	Est. Population living on less than $2 per day [12], [13]
Bolivia	10.6 million	24.9% (2008)	2.6 million
Cuba	11.0 million	No information provided	-
Ecuador	15.6 million	10.6% (2010)	1.6 million
Nicaragua	6.1 million (2012)	31.7% (2005)	1.8 million
Venezuela	28.9 million	12.9% (2006)	3.7 million
Total in Bolivia, Ecuador, Nicaragua, and Venezuela (Cuba excluded)	71.9 million		9.7 million
Total in LAC region	581 million		99 million
% in the five countries	12%		10%

NTDs are commonly found wherever poverty is pervasive [12], and as shown in Table 2, the LAC region's major NTDs—Chagas disease, cutaneous leishmaniasis (CL), dengue, intestinal helminth infections, and malaria (mostly vivax malaria)—are highly endemic in Bolivia, Ecuador, Nicaragua, and Venezuela, while dengue is also an important NTD in Cuba [14]–[19]. Approximately 14–15 percent of the cases of these NTDs occur in Bolivia, Ecuador, Nicaragua, and Venezuela, despite the fact that these countries only comprise about 10% of the population. Of these countries, Bolivia leads in the number of Chagas disease cases (620,000) and has the highest prevalence of this disease anywhere in the LAC region, Nicaragua leads in cutaneous leishmaniasis cases (9,000–14,800), and Venezuela has the largest number of dengue fever cases (3.5 million) [14]–[16]. The greatest number of children who require deworming for intestinal helminth infections also live in Bolivia (3.4 million), and Ecuador leads in terms of its population at risk for malaria (mostly vivax malaria) with 6.6 million [17]–[19]. Even though Cuba is far better off economically, it too has a large number of cases of dengue fever and intestinal helminth infections [16]–[18]. Thus, a significant percentage of the people of Bolivia, Cuba, Ecuador, Nicaragua, and Venezuela have at least one NTD.

**Table 2:** Neglected tropical disease cases in five Latin American countries.

Country	Chagas disease [14]	Cutaneous Leishmaniasis [15]	Dengue fever [16]	Intestinal helminth infections [17], [18]	Malaria (Population at risk in 2011) [19]
Bolivia	620,000	7,400 to 12,200	181,219 apparent, 555,702 inapparent, 736,921 total	984,149 preschool, 2,377,426 SAC*, 3,361,575 total	1,321,178
Cuba	Non-endemic	Non-endemic	372,825 apparent, 1,132,115 inapparent, 1,504,940 total	39,626 preschool, 122,381 SAC*, 162,007 total	Malaria eliminated
Ecuador	230,000	4,800 to 7,900	310,448 apparent, 951,375 inapparent, 1,261,823 total	91,935 preschool 229,480 SAC*, 321,415 total	6,569,649
Nicaragua^1^	58,600^1^	9,000 to 14,800^1^	172,439 apparent, 526,486 inapparent, 698,925 total^1^	545,819 preschool, 1,301,128 SAC*, 1,846,947 total^1^	2,575,374^1^
Venezuela	310,000	6,900 to 11,400	866,172 apparent, 2,634,742 inapparent, 3,500,914 total	280,283 preschool, 676,481 SAC*, 956,764 total^1^	5,705,160^1^
Total in five countries	1.2 million	28,100 to 46,300	7,703,523 total	6,648,708	16,171,361
Total in LAC region	7.8 million	187,200 to 307,800	13.3 million apparent, 40.5 million inapparent, 53.8 million total	48 million	106,469,796
% in the five countries	15%	15%	14%	14%	15%

*School-aged children.

1 The data cited here on Nicaragua also appears in a paper under review for a special ICOPA XIII issue of the International Journal for Parasitology: Hotez PJ, Woc-Colburn L, Bottazzi ME. Neglected Tropical Diseases in Central America and Panama: Review of their Prevalence, Populations at Risk, and Impact on Regional Development. Int J Parasitol.

Such high numbers of people affected by NTDs afford potential opportunities for the US to work with these countries in programs of science and global health diplomacy [20]. These programs might include bilateral cooperative efforts to implement disease control and elimination programs for Chagas disease, CL, dengue, intestinal helminth infections, and malaria, potentially relying on shared expertise between the US and the disease-endemic countries. An example is the dengue fever epidemiological research program in Nicaragua led for over two decades by Dr. Eva Harris at the University of California Berkeley [21]. Moreover, at least three of these NTDs—Chagas disease, CL, and dengue—have also emerged in the southern US, especially in Texas and some other areas on the Gulf Coast [22], so the US might also actually benefit from the public health expertise of the five LAC countries highlighted here. In some areas of disease control, the US could work jointly with Cuba to tackle NTD endemicity in the four low-income LAC countries.

There may also be specific opportunities for “vaccine diplomacy,” which I defined previously as a form of science diplomacy focused on “joint development of life-saving vaccines and related technologies” conducted by scientists from “nations that often disagree ideologically” or even those “actively engaged in hostile actions” [23]. Today, of the countries discussed here, both the US and Cuba stand out for their programs of vaccine research and development (R&D), with Cuba's Instituto Finlay, for instance, belonging to the renowned Developing Countries Vaccine Manufacturers Network [24]. Joint US–Cuba programs in NTD vaccines, possibly including scientists from Bolivia, Ecuador, Nicaragua, or Venezuela, offer additional mechanisms on this front. Other LAC countries with relevant vaccine development capabilities include Brazil (primarily FIOCRUZ Bioamanguinhos and Instituto Butantan), Mexico (Birmex), and Argentina [24]. Also in Venezuela, a group at the Instituto de Biomedicina of Universidad Central de Venezuela in Caracas previously developed a therapeutic vaccine for CL containing heat-killed pasteurized *Leishmania* promastigotes together with Bacillus Calmette-Guerin (BCG), as well as other innovative therapies [25], [26]. Nonprofit product development partnerships (PDPs), including our Sabin Vaccine Institute and Texas Children's Hospital Center for Vaccine Development, could play key roles in fostering ties with these developing country vaccine manufacturers [27].

Overall, science and global health diplomacy as it pertains to NTDs in Latin America comprise a modest element of today's US foreign policy. Currently USAID's NTD Program does not include any of the countries highlighted here [28], and the President's Malaria Initiative (PMI) does not operate in Latin America [29]. However, Nicaragua is listed as a PEPFAR (US President's Emergency Plan for AIDS Relief) country [30], while both Nicaragua and Ecuador are listed among the important nations where USAID operates [31]. On the Cuban side, for at least fifty years medical diplomacy has been central to Cuba's outreach to the LAC region, but it is not clear which, if any, of these activities pertain to the NTDs [32].

Ultimately it could be exciting to see how joint programs of NTD control and vaccine and other types of R&D might become front and center to US foreign policy towards Latin America. Such programs represent an important, potentially highly productive, and yet largely untapped opportunity.

## References

[1] Barshefsky C, Hill JT, O'Neil SK, Sweig JE, for the Council on Foreign Relations (2008) U.S.-Latin America Relations: A New Direction for a New Reality. Independent Task Force Report No. 60. New York: Council on Foreign Relations Press. Available: http://www.cfr.org/mexico/us-latin-america-relations/p16279. Accessed 25 August 2014.

[2] US Department of State (2013) US Relations with Bolivia. Fact sheet Available: http://www.state.gov/r/pa/ei/bgn/35751.htm. Accessed 15 January 2014.

[3] RT (2013) Latin America no longer “US' backyard”— Ecuadorian Foreign Minister. Available: http://rt.com/news/ecuador-us-relations-spying-908/. Accessed 16 January 2013.

[4] US Department of State (2013) US Relations with Ecuador. Fact sheet Available: http://www.state.gov/r/pa/ei/bgn/35761.htm. Accessed 18 January 2014.

[5] Ullman R (1983) At War with Nicaragua. Foreign Affairs, Fall 1983 Available: http://www.foreignaffairs.com/articles/37968/richard-h-ullman/at-war-with-nicaragua. Accessed 19 January 2014.

[6] US Department of State (2014) US Relations with Nicaragua. Fact sheet Available: http://www.state.gov/r/pa/ei/bgn/1850.htm. Accessed 18 January 2014.

[7] US Department of State (2013) US Relations with Venezuela. Fact sheet Available: http://www.state.gov/r/pa/ei/bgn/35766.htm. Accessed 18 January 2014.

[8] Baverstock A, Strange H (6 October 2013) As socialist dream crumbles, Venezuelans find Nicolas Maduro ‘a bad copy’ of Chavez. The Telegraph Available: http://www.telegraph.co.uk/news/worldnews/southamerica/venezuela/10359267/As-socialist-dream-crumbles-Venezuelans-find-Nicolas-Maduro-a-bad-copy-of-Chavez.html. Accessed 4 January 2014.

[9] US Department of State (2013) US Relations with Cuba. Fact sheet Available: http://www.state.gov/r/pa/ei/bgn/2886.htm.

[10] The World Bank (2014) Data: Latin America & Caribbean (developing only). Available: http://data.worldbank.org/region/LAC. Accessed 5 January 2014.

[11] Central Intelligence Agency (2014) World Factbook. Available: https://www.cia.gov/library/publications/the-world-factbook/. Accessed 26 August 2014.

[12] Hotez PJ, Dumonteil E, Heffernan MJ, Bottazzi ME (2013) Innovation for the ‘bottom 100 million’: eliminating neglected tropical diseases in the Americas. Adv Exp Med Biol 764: 1–12. 23654053 10.1007/978-1-4614-4726-9_1

[13] Wikipedia (2014) List of Countries by percentage of population living in poverty. Available: http://en.wikipedia.org/wiki/List_of_countries_by_percentage_of_population_living_in_poverty. Accessed 18 January 2014.

[14] Bern C, Kjos S, Yabsley MJ, Montgomery SP (2011) Trypanosoma cruzi and Chgas' disease in the United States. Clin Microbiol Rev 24: 655–681. 21976603 10.1128/CMR.00005-11 PMC3194829

[15] Alvar J, Velez ID, Bern C, Herrero M, Desjeuz P, et al (2012) Leishmaniasis worldwide and global estimates of its incidence. PLoS ONE 7: e35671. 22693548 10.1371/journal.pone.0035671 PMC3365071

[16] Bhatt S, Gething PW, Brady OJ, Messina JP, Farlow AW, et al (2013) The global distribution and burden of dengue. Nature 496: 504–507. 23563266 10.1038/nature12060 PMC3651993

[17] World Health Organization (2014) Neglected tropical diseases, PCT Databank. Soil-transmitted helminthiases Available: http://www.who.int/neglected_diseases/preventive_chemotherapy/sth/en/index.html. Accessed 14 January 2014.

[18] World Health Organization (2013) Soil-transmitted helminthiases: number of children treated in 2011. Weekly Epidemiol Rec 88: 145–152. 23586139

[19] Pan American Health Organization (2011) Report on the Situation of Malaria in the Americas, 2011. Available: http://www.paho.org/hq/index.php?option=com_content&view=article&id=2459&Itemid=2000&lang=en. Accessed 2 January 2014.

[20] Katz R, Kornblet S, Arnold G, Lief E, Fischer JE (2011) Defining health diplomacy: changing demands in the era of globalization. Milbank Q 89: 503–523. 21933277 10.1111/j.1468-0009.2011.00637.x PMC3214719

[21] UC Global Health Institute (2014) Eva Harris, PhD. Available: http://www.ucghi.universityofcalifornia.edu/news-events/profiles/harris-eva.aspx. Accessed 4 January 2014.

[22] Hotez PJ, Murray KO, Buekens P (2014) The Gulf Coast: a new American underbelly of tropical diseases and poverty. PLoS Negl Trop Dis 8: e2760. 24830815 10.1371/journal.pntd.0002760 PMC4022458

[23] Hotez PJ (2014) “Vaccine diplomacy”: historical perspectives and future directions. PLoS Negl Trop Dis 8: e2808. 24968231 10.1371/journal.pntd.0002808 PMC4072536

[24] DCVMN (2014) Developing Countries Vaccine Manufacturers Network. Available: http://www.dcvmn.org/. Accessed 3 January 2014.

[25] Convit J, Ulrich M, Zerpa O, Borges R, Aranzazu N, et al (2003) Immunotherapy of american cutaneous leishmaniasis in Venezuela during the period 1990–99. Trans R Soc Trop Med Hyg 97: 469–472. 15259484 10.1016/s0035-9203(03)90093-9

[26] Convit J, Ulrich M, Argelia Polegre M, Avila A, Rodriguez N, et al (2004) Therapy of Venezuelan patients with severe mucocutaneous or early lesions of diffuse cutaneous leishmaniasis with a vaccine containing pasteurized *Leishmania* promastigotes and Bacillus Calmette-Guerin – preliminary report. Mem Inst Oswaldo Cruz 99: 57–62. 15057348 10.1590/s0074-02762004000100010

[27] Hotez P (2011) A handful of ‘antipoverty’ vaccines exist for neglected diseases, but the world's poorest billion people need more. Health Aff (Millwood) 30: 1080–1087. 21653960 10.1377/hlthaff.2011.0317

[28] USAID's NTD program (2014) Countries supported by USAID's NTD Program. Available: http://www.neglecteddiseases.gov/countries/index.html. Accessed 5 January 2014.

[29] President's Malaria Initiative (2014) Fighting Malaria and Saving Lives. Available: http://www.pmi.gov/about. Accessed 26 August 2014.

[30] The United States President's Emergency Plan for AIDS Relief (2014) Countries. Available: http://www.pepfar.gov/countries/index.htm. Accessed 11 January 2014.

[31] USAID (2014) Latin America and the Caribbean. Available: http://www.usaid.gov/where-we-work/latin-american-and-caribbean. Accessed 5 January 2014.

[32] Feinsilver JM (2010) Fifty years of Cuba's medical diplomacy: from idealism to pragmatism. Cuban Stud 41: 85–104. 21506308

